# A CUG codon-adapted anchor-away toolkit for functional analysis of genes in *Candida albicans*

**DOI:** 10.1128/msphere.00703-23

**Published:** 2024-01-22

**Authors:** Basharat Bashir Teli, Priyanka Nagar, Yumnam Priyadarshini, Poonam Poonia, Krishnamurthy Natarajan

**Affiliations:** 1 Laboratory of Eukaryotic Gene Regulation, School of Life Sciences, Jawaharlal Nehru University, New Delhi, India; University of Georgia, Athens, Georgia, USA

**Keywords:** *Candida albicans*, functional genomics, anchor-away, essential genes, non-essential genes, *TBP1*, SPT8

## Abstract

Promoter shutoff of essential genes in the diploid *Candida albicans* has often been insufficient to create tight, conditional null alleles due to leaky expression and has been a stumbling block in pathogenesis research. Moreover, homozygous deletion of non-essential genes has often been problematic due to the frequent aneuploidy in the mutant strains. Rapid, conditional depletion of essential genes by the anchor-away strategy has been successfully employed in *Saccharomyces cerevisiae* and other model organisms. Here, rapamycin mediates the dimerization of human FK506-binding protein (FKBP12) and FKBP12-rapamycin-binding (FRB) domain-containing target protein, resulting in relocalization to altered sub-cellular locations. In this work, we used the ribosomal protein Rpl13 as the anchor and took two nuclear proteins as targets to construct a set of mutants in a proof-of-principle approach. We first constructed a rapamycin-resistant *C. albicans* strain by introducing a dominant mutation in the *CaTOR1* gene and a homozygous deletion of *RBP1*, the ortholog of *FKBP12*, a primary target of rapamycin. The *FKBP12* and the *FRB* coding sequences were then CUG codon-adapted for *C. albicans* by site-directed mutagenesis. Anchor-away strains expressing the essential *TBP1* gene or the non-essential *SPT8* gene as *FRB* fusions were constructed. We found that rapamycin caused rapid cessation of growth of the TBP-AA strain within 15 minutes and the SPT8-AA strain phenocopied the constitutive filamentous phenotype of the *spt8*Δ/*spt8*Δ mutant. Thus, the anchor-away toolbox for *C. albicans* developed here can be employed for genome-wide analysis to identify gene function in a rapid and reliable manner, further accelerating anti-fungal drug development in *C. albicans*.

**IMPORTANCE:**

Molecular genetic studies thus far have identified ~27% open-reading frames as being essential for the vegetative growth of *Candida albicans* in rich medium out of a total 6,198 haploid set of open reading frames. However, a major limitation has been to construct rapid conditional alleles of essential *C. albicans* genes with near quantitative depletion of encoded proteins. Here, we have developed a toolbox for rapid and conditional depletion of genes that would aid studies of gene function of both essential and non-essential genes.

## INTRODUCTION

*Candida albicans* is the most prevalent opportunistic fungal pathogen in humans. *C. albicans* causes bloodstream infections, ranging from superficial/mucosal and deep-seated/systemic infections in humans, particularly in immune-compromised individuals (1). As drug resistance is a major threat, there is an urgent need to identify new genes/pathways that are essential for the survival and pathogenicity of *C. albicans*. In addition to the essential genes, studies have shown that genes required for critical pathways, including iron homeostasis (2, 3), ergosterol biosynthesis (4), and fatty acid biosynthesis (5), are also known to be important for survival in the host.

The budding yeast *Saccharomyces cerevisiae* ORFs have been extensively studied using a focused, gene-based approach as well as through genome-wide studies to understand gene function (6–8). Despite the advances made in functional analysis of genes in the post-genome era, ~12% of the budding yeast genes remain uncharacterized (9). Moreover, ~1100 ORFs are essential for vegetative growth of *S. cerevisiae*, and nearly 40% of the *C. albicans* genes do not seem to have an ortholog in *S. cerevisiae* (10). Furthermore, as *C. albicans* is diploid and without a sexual cycle, classical genetic approaches used for *S. cerevisiae* cannot be used. As null mutations cannot be constructed for essential genes, transcriptional repression has been employed in *C. albicans* (11–13). However, inactivation by depletion has suffered from either a long delay in depletion with attendant secondary effects or incomplete depletion due to leaky promoter shutoff. Therefore, an efficient strategy known as the anchor-away technique has been developed to relocate nuclear proteins to cytoplasmic ribosomes, resulting in rapid depletion of target proteins (14).

**Fig 1:**
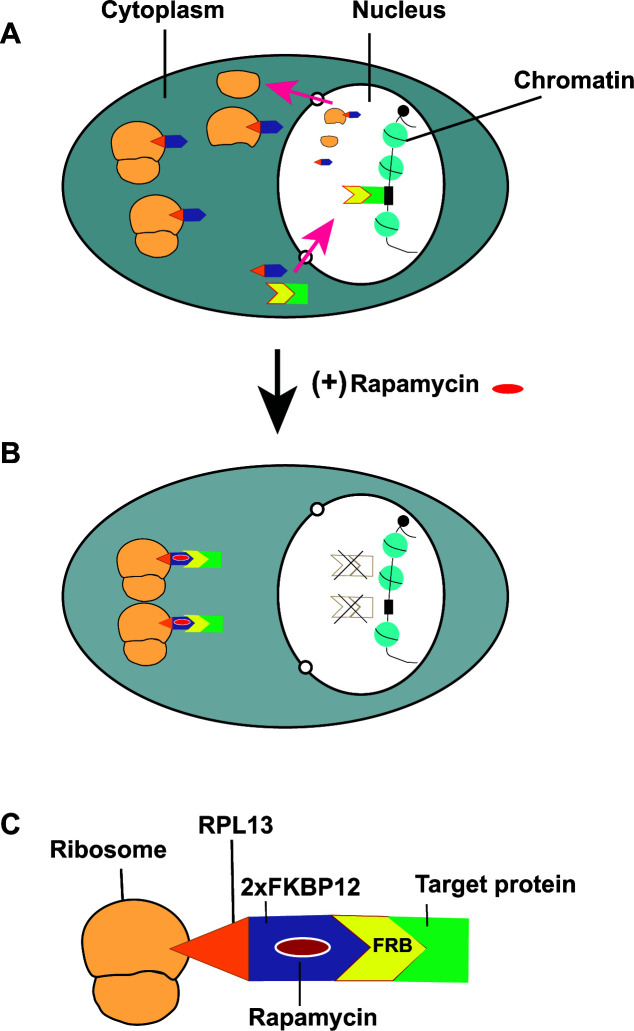
Schematic diagram depicting the anchor-away technique. (**A**) Under normal growth conditions, the ribosomal proteins are transported into the nucleus (red arrow) for the assembly of large and small ribosomal subunits. The large subunit including Rpl13-FKBP12 and the small subunits would be exported to the cytoplasm, and ribosomes are formed. Moreover, the FRB-tagged candidate nuclear protein would be chromatin-bound. (**B**) Upon rapamycin addition, the FRB-tagged candidate nuclear protein would be tethered to ribosomes in the cytoplasm by rapamycin-mediated interaction of FRB-FKBP12 domains. (**C**) Molecular components of the anchor-away system. Here, a ribosome is shown to contain the FKBP12-tagged Rpl13 that has sequestered the target protein through the rapamycin-mediated interaction of FRB-FKBP12 domains.

In the anchor-away technique (AAT), an abundant cytoplasmic ribosomal subunit protein such as L13, a 60S subunit, is C-terminally tagged with two tandem copies of a segment of FKBP12, and the protein of interest is tagged with the FRB domain of Tor1 (15) and expressed as fusion proteins. Studies on ribosome biogenesis (16, 17) have shown that under normal growth conditions, ribosomal proteins along with their assembly factors from the cytoplasm are imported into the nucleus for ribosomal subunit maturation incorporating rRNA components in the nucleolus, and the mature large (60S) and small (40S) ribosomal subunits are exported back into the cytoplasm (Fig. 1A). The Rpl13A-2×FKBP12 fusion protein (called anchor) would then be integrated into the large ribosomal subunit as they exit the nucleus (Fig. 1A). In the presence of rapamycin, however, the protein of interest expressed as a fusion protein with the FRB domain is tethered onto the 60S subunit through an interaction between FRB and FKBP12 domains (Fig. 1A), thereby sequestering the target nuclear protein into ribosome particles in the cytoplasm. The target nuclear protein would then become unavailable in the nucleus, thereby creating loss-of-function mutants (Fig. 1B). Studies employing the AAT in *S. cerevisiae* have demonstrated the utility of this technique for gene inactivation and functional studies of several different genes (18–24). The Rpl13A-FKBP12/FRB anchor-away system, although widely used to study regulatory nuclear proteins, has also been successful in the depletion of cytoplasmic or membrane proteins (25, 26). Moreover, the FKBP12/FRB anchor-away system has also been used to relocate Sec21-FRB to OM45-FKBP12 in the mitochondria (27), thereby indicating the broad scope of the AAT system for gene inactivation studies. The anchor-away technique has also been developed for *Schizosaccharomyces pombe* (28), *Drosophila* (29), and human cells (30).

In this study, we have constructed *C. albicans* anchor-away strains for functional analysis of genes. Studies on the budding yeast have uncovered genetic and physical interactions between Spt8 and TBP and demonstrated their critical roles in transcriptional regulation (31–34). By employing this system, we generated anchor-away strains for *TBP1* and *SPT8* candidate genes, and their phenotypic characterization revealed that our AAT is a rapid and effective system and thus highly suited to deplete essential as well as non-essential genes in *C. albicans*.

## RESULTS

### Adapting the anchor-away system to *C. albicans*

**Fig 2:**
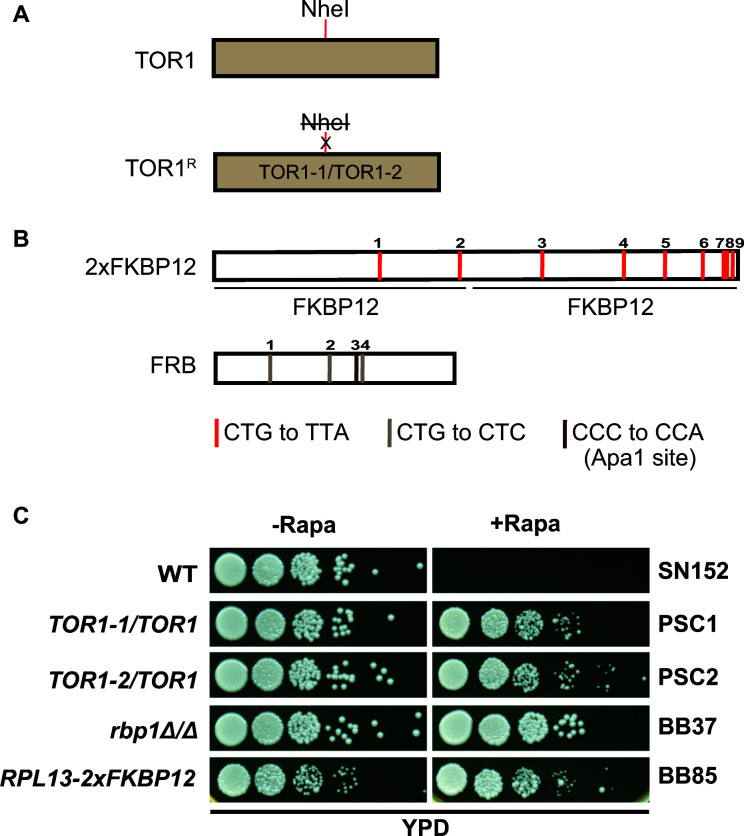
Adapting the anchor-away system to *C. albicans*. (**A**) Schematic diagram showing the wild-type *TOR1* and the dominant mutants *TOR1-1* and *TOR1-2* and the presence/absence of the NheI site. (**B**) Schematic diagram showing the location of mutagenized CTG codons constructed by site-directed mutagenesis. The nine CTG codons in 2×FKBP12 were each mutagenized to the TTA codon and to the three CTG codons in the *FRB* sequence; an ApaI site was created in the FRB sequence by site-directed mutagenesis. (**C**) Growth phenotypes of *C. albicans* strains *TOR1-1* (PSC1)*, TOR1-2* (PSC2), and *rbp1*Δ/*rbp1*Δ (BB37) and the base strain *RPL13A-2×FKB12* (BB85) were assessed by growth in yeast extract-peptone-dextrose (YPD) plates with 1 µg/mL rapamycin (+Rapa) or control dimethyl sulfoxide (DMSO) (-Rapa) at 30°C for 36 hours, and the images were acquired.

To create anchor-away strains, we first constructed a rapamycin-resistant *C. albicans* base strain in the wild-type SN152 background (35). Towards this end, we introduced site-directed mutation in the FRB domain of *C. albicans TOR1* as reported previously (36). We introduced one of the two dominant mutations Ser1984Arg (*TOR1-1*) or Ser1984Ile (*TOR1-2*) in the *C. albicans* SN152 strain (Fig. 2A) in independent transformation reactions. Rapamycin-resistant transformants were selected, genomic DNA isolated, *TOR1* sequence PCR-amplified, and the *TOR1-1* or *TOR1-2* candidates were screened for the presence of the respective *TOR1* mutations by NheI restriction digestion to identify *TOR1-1/TOR1-2* mutants (no NheI) from the wild-type *TOR1* strain (with NheI site), thereby providing an assay to screen the candidate mutants (36).

To avoid potential competitive binding of rapamycin to Rbp1, the *C. albicans* homolog of the *S. cerevisiae FPR1*, encoding the ortholog of the mammalian FK506-binding protein, we constructed a homozygous *rbp1*Δ/*rbp1*Δ mutant strain (see Materials and Methods). The homozygous deletion was confirmed by PCR.

*C. albicans* belongs to the CTG clade of fungi and decodes the CUG codon as serine and not as leucine. The *2×FKBP12* sequence and the *FRB* sequence in plasmids p30582 and p30579 (14), respectively, contained multiple CTG codons. Therefore, site-directed mutagenesis was carried out using long oligos to replace the nine CTG codons in *2×FKBP12* to TTA codons by PCR to amplify *2×FKBP12* in three overlapping fragments by using p30582 as a template (Fig. 2B). The three fragments were then fused by overlap extension PCR (37), followed by blunt-end cloning into the Ip27 plasmid to obtain plasmid YPC36, and the sequence of the mutagenized *FKBP12* region was confirmed by DNA sequencing (see Fig. S1 at https://www.jnu.ac.in/Faculty/natarajan/data.htm). To replace the three CTG triplets in the *FRB* coding sequence to CTC, long oligos were used for Klenow extension as two overlapping DNA fragments, which were then fused by overlap extension PCR. An ApaI site was also introduced in the FRB fragment to allow screening of mutant clones (Fig. 2B). Next, overlap extension PCR was used to fuse the fragments followed by blunt-end cloning into pSN52 and pSN69 (38) vectors, and plasmids pBB2 and pBB8 were obtained. The sequence of the mutagenized *FRB* region was confirmed by DNA sequencing (see Fig. S2 at https://www.jnu.ac.in/Faculty/natarajan/data.htm).

The *C. albicans* genome contains a single essential ORF C1_03020C/19.2994 encoding the large ribosomal subunit protein L13/Rpl13. As Rpl13a was used as an anchor protein in *S. cerevisiae* (14), *S. pombe* (28), and *Drosophila* (29), we selected C1_03020C/*RPL13* encoding L13/Rpl13 to build our anchor-away strain. The *RPL13* ORF was tagged at the C-terminus with two copies of CUG codon-adapted *FKBP12* amplified from plasmid pBB43 by integration at the genomic locus, and the correct integration was confirmed by PCR (39).

To test the growth of strains on rapamycin, the *C. albicans* strains *TOR1-1*, *TOR1-2*, and *rbp1*Δ/*rbp1*Δ, the 2×FKBP12-tagged base strain (BB85), and the wild-type control strain SN152 were pre-grown in YPD and serially diluted to fresh medium, and aliquots were spotted on YPD plates containing rapamycin or vehicle control DMSO. The spot assay results showed that the wild-type control strain SN152, as expected, did not grow on rapamycin, while all other strains were able to grow in the presence of rapamycin, although the base strain BB85 had mild growth defect compared to the parental strains (Fig. 2C). Thus, we built the rapamycin-resistant strain BB85 as the base strain bearing *RPL13-2×FKBP12* for generating anchor-away alleles encoding different target proteins.

### Construction of *TBP1-AA* and *SPT8-AA* strains

As a proof of principle for setting up the anchor-away system in *C. albicans*, we chose two nuclear-localized proteins, viz., *TBP1/SPT15* (essential) and *SPT8* (non-essential). TBP is a general transcription initiation factor that plays a central role in transcription at eukaryotic promoters (40, 41). Therefore, tethering TBP onto ribosomes in the cytoplasm is expected to confer a lethal phenotype, as shown for *S. cerevisiae* (14, 42). Spt8 is a subunit of the SAGA (Spt-Ada-Gcn5-acetyltransferase) multiprotein co-activator complex involved in TBP recruitment to gene promoters in yeast (31–33, 43).

**Fig 3:**
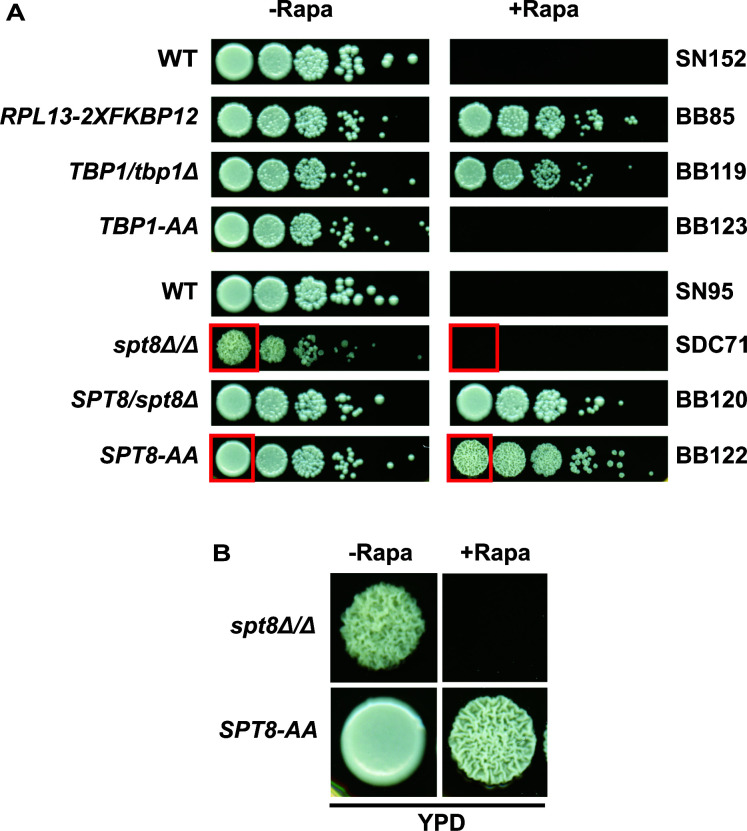
Phenotypic screening of *TBP1-AA* and *SPT8-AA* strains. (**A**) *C. albicans* strains SN152, BB85, BB119, and BB123 and *C. albicans* strains SN95, SDC71, BB120, and BB122 were grown overnight in YPD plates to saturation, serially diluted, and 5-µl dilutions (10-fold) were spotted onto YPD (-Rapa) or with rapamycin (1 µg/mL; +Rapa) plates, grown at 30°C for 48 hours, and imaged. (**B**) The images marked with a red box in panel A are shown as a zoomed image to display the rough colony morphology of strains SDC71 (*spt8*Δ/Δ) and BB122 (SPT8-AA).

To construct anchor-away strains, we first produced heterozygous deletion strains followed by tagging the second allele with the *FRB* sequence in-frame to the 3’end of the ORF using the PCR-mediated strategy (12). To this end, we prepared a heterozygous deletion strain of *TBP1* (BB119) and *SPT8* (BB120) in the BB85 genetic background marked with *C. maltosa LEU2* amplified from the pSN40 (38) plasmid. The second alleles of *TBP1* and *SPT8* were then tagged at their C-terminal ends with the codon-adapted *FRB* tag amplified from plasmid pBB30. The correct integration of the *FRB* tag was confirmed by PCR. Next, we tested the phenotype of the *SPT8-AA* (BB122) and *TBP1-AA* (BB123) strains on YPD plates with or without rapamycin by spot assays. The results showed that the *TBP1-AA* strain demonstrated a lethal phenotype in the presence of rapamycin compared to the control plate (without rapamycin), indicating that sequestration of the TBP-FRB protein onto ribosomes rendered the cells inviable (Fig. 3A). As a control, we also tested *S. cerevisiae TBP1-AA* strain KHW76 in comparative experiments along with *C. albicans TBP1-AA* strain BB123 and showed that the growth phenotypes of the *TBP1-AA C. albicans* (Fig. 3) and *S. cerevisiae* (see Fig. S3 at https://www.jnu.ac.in/Faculty/natarajan/data.htm) strains are comparable in the rapamycin-containing medium.

Next, we tested the phenotype of our *C. albicans SPT8-AA* strain and found it to be viable even in the presence of rapamycin. However, rapamycin induced a marked rough colony morphology, indicative of a filamentous phenotype (Fig. 3A and B). We also compared the growth properties of the *SPT8-AA* strain with the *spt8*Δ/*spt8*Δ mutant. Indeed, the rough colony morphology of the *spt8*Δ/*spt8*Δ mutant in the YPD medium was phenocopied by the *SPT8-AA* strain in the presence of rapamycin but not in the control plate without rapamycin (Fig. 3A and B). These results demonstrate that the AAT efficiently works in *C. albicans* to induce a loss-of-function phenotype.

### Steady-state protein levels of anchor (Rpl13-2×FKBP12) and target fusion (Spt8-FRB and TBP-FRB) proteins

**Fig 4:**
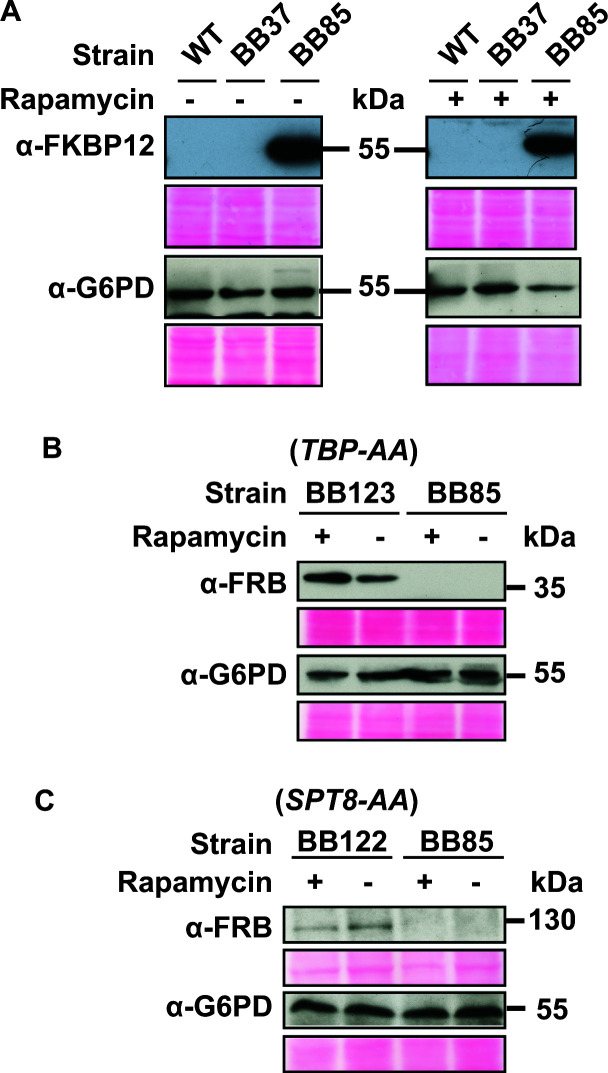
Expression of anchor protein (Rpl13-2×FKBP12) and the target Spt8-AA and TBP-AA fusion proteins. (**A**) *C. albicans* strains SN152, BB37, and BB85 were grown in YPD medium overnight, diluted in fresh YPD medium, and treated with rapamycin (1 µg/mL; +Rapa) or control DMSO (-Rapa) for 1 hour; cells were harvested and protein lysates were prepared. About ~200 µg total protein was resolved in SDS-polyacrylamide gel electrophoresis (SDS-PAGE), blotted to nitrocellulose membranes, and immunoblotted with anti-FKBP12 antibody, and proteins were detected. (**B**) *C. albicans* strains BB123 (TBP-AA) and control BB85 and (**C**) *C. albicans* strains BB122 (SPT8-AA) and control BB85 strains were similarly analyzed by Western blotting and probed with anti-FRB antibody. Anti-glucose 6-phosphate dehydrogenase (G6PD) antibody was used as the loading control.

To check the expression level of the anchor fusion protein Rpl13-2×FKBP12 in *C. albicans* strains SN152, BB37, and BB85 (*RPL13-2×FKBP12*), the cultures were treated with 1 µg/mL of rapamycin for 1 hour, cells harvested, and protein lysates prepared (see Materials and Methods). The protein levels before and after rapamycin treatment were assessed by immunoblotting using anti-FKBP12 antibody. Whole cell extracts were prepared from cultures treated or not treated with rapamycin, and Western blot was conducted. The Western blot results showed that there were no significant effects on the steady-state protein level of the anchor protein after 1 hour exposure to rapamycin with reference to the control G6PD (Fig. 4A). The levels of Spt8-FRB and TBP*-*FRB fusion proteins were also analyzed in the *C. albicans* BB85, BB122 (*SPT8-AA*), and BB123 (*TBP1-AA*) strains. By using an anti-FRB antibody for immunoblotting, the protein levels of the anchor-away strains were compared before and after 1 hour of rapamycin treatment, and it was found that there were no significant effects on the steady-state protein levels, as seen in the case of the expression of the anchor protein as well (Fig. 4B and C). As antibodies against FRB and were used here, the effect of the tags, if any, on the protein expression level or stability of the Rpl13 anchor or the target proteins could not be determined. The *S. cerevisiae* TBP-AA strain (KHW76) (42) also expressed the ScRpl13a-2×FKBP12 fusion protein under both minus and plus rapamycin conditions (see Fig. S3 at https://www.jnu.ac.in/Faculty/natarajan/data.htm). Thus, the results demonstrate that the loss-of-function phenotypes shown by *SPT8-AA* (filamentous) and *TBP1-AA* (lethal) strains are due to sequestering of the respective fusion proteins in the cytoplasm but not due to degradation of the fusion proteins.

### Characterization of growth pattern and viability of anchor-away strains

**Fig 5:**
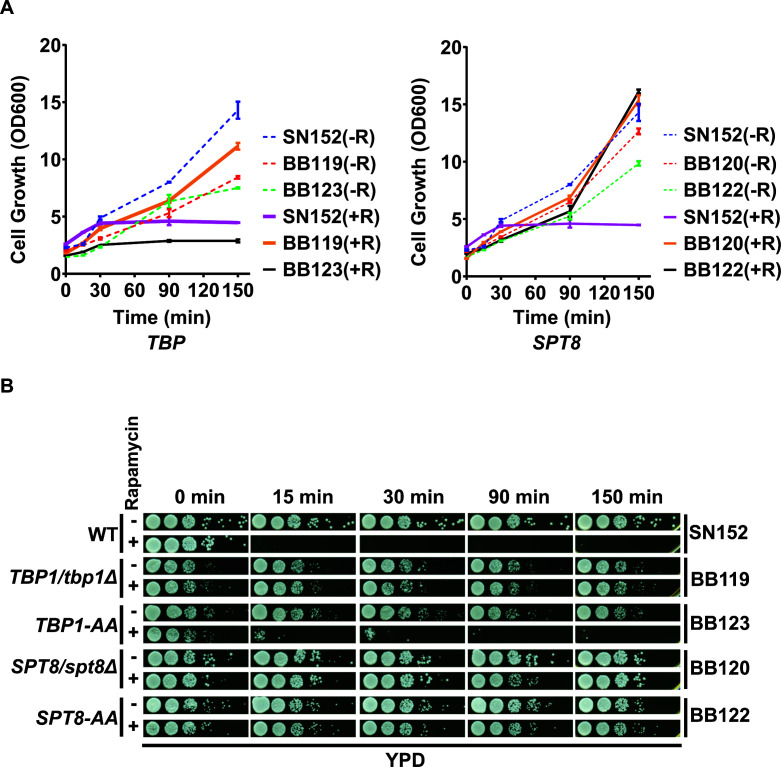
Rapid manifestation of the phenotype upon anchor-away. *C. albicans* strains SN152, BB119 (*TBP1/tbp1*Δ), BB123 (*TBP-AA*), BB120 (*SPT8/spt8*Δ), and BB122 (*SPT8-AA*) were grown in YPD medium overnight, diluted in fresh YPD medium with 1 µg/mL rapamycin (+Rapa) or with DMSO vehicle control (-Rapa), and grown for indicated time periods, and (**A**) cell growth measured at OD_600_ or (**B**) cells harvested, washed, and spotted in the YPD medium and incubated at 30°C.

A liquid growth assay was performed to assess the growth of all the parental and anchor-away strains in the presence and absence of rapamycin. The wild-type (SN152), parental (BB120 or BB119), and the anchor-away strains (BB122 or BB123) cultured in YPD medium without rapamycin, as expected, showed comparable growth (Fig. 5A and B). In the presence of rapamycin, however, the parental strains BB120 or BB119 and the *SPT8-AA* strain BB122 showed robust growth (Fig. 5A and B). The *TBP-AA* strain BB123, however, rapidly ceased to grow in the presence of rapamycin (Fig. 5A). The wild-type control strain SN152, as expected, could not grow in the rapamycin-containing medium (Fig. 5A and B).

To test how rapidly the *TBP-AA* strain lost viability, we harvested cells grown in YPD medium with or without rapamycin for different time intervals, washed, serially diluted, and carried out spot assays on YPD plates at 30°C for 36 hours. The spot assay results showed that the *TBP-AA* strain lost substantial viability even by 15 minutes of rapamycin treatment in the culture, and by 90 minutes, little or no viable cells could be recovered, indicating a rapid cessation of growth upon targeting of TBP onto ribosomes with an attendant lethal phenotype (Fig. 5B). The *SPT8-AA* strain, however, grew as smooth colonies on the YPD plate comparable to the wild-type strain SN152 or the *SPT8-AA/SPT8* heterozygous strain BB120 even after 150 minutes of rapamycin exposure (Fig. 5B), unlike the rough colony morphology of the *SPT8-AA* strain obtained only upon prolonged exposure on YPD plus rapamycin plates (Fig. 3B). The phenotype of the *SPT8-AA* strain suggested that the anchor-away technique can also restore the growth defect upon return to normal growth conditions, suggesting reversibility of the *SPT8-AA* strain.

## DISCUSSION

Genetic analysis of *C. albicans* genes has been majorly limited to the study of the function of non-essential genes using homozygous null mutant strains. As null mutants cannot be constructed for essential genes and null mutations in certain non-essential genes also lead to aneuploid states, a robust inactivation technique that results in rapid depletion (relocating) of candidate proteins is required for studying gene functions. Although transcriptional repression has been widely employed in *C. albicans*, leaky expression or incomplete depletion has been a significant limitation for the quantitative inactivation of candidate genes.

To circumvent these limitations, the AAT was first developed and widely used in *S. cerevisiae* to deplete proteins by sequestering them to ribosomes in the cytoplasm (14). In this work, we have created a toolbox for the AAT by CTG codon adaptation for *C. albicans,* leading to effective depletion of the target protein, which resulted in inactivation within ≤1 hour after rapamycin addition. Since rapamycin is toxic to wild-type *C. albicans,* we generated *TOR1* and *RBP1* mutants and made cells rapamycin-resistant. *C. albicans* belongs to the CTG group of fungi (44), and therefore, to use *2×FKBP12* and *FRB* tags of human origin in *C. albicans*, we optimized their CTG codons so as to code for leucine instead of serine in *C. albicans*. The plasmids containing CTG codon-optimized *2×FKBP12* (pBB43) and *FRB* (pBB30) sequences were constructed, and the candidate genes were tagged in the anchor-away base strain (BB85). We observed that the *RPL13-2×FKBP12* base strain showed a mild growth defect, possibly a result of tagging the *RPL13* gene. We chose *SPT8* (non-essential) and *TBP1* (essential), two nuclear proteins, as proof of concept for the *C. albicans* anchor-away technique. These anchor-away strains were tested for the effect of depletion on YPD-rapamycin plates, wherein the *SPT8-AA* strain (BB122) showed a filamentous phenotype comparable to that of the *spt8*Δ*/spt8*Δ mutant, and the *TBP-AA* strain (BB123) showed a lethal phenotype, as expected. Western blot analysis of the anchor-away strains confirmed that anchor and target fusion proteins showed a significant level of expression even after 1-hour exposure to rapamycin (Fig. 4A through C), indicating that the mutant phenotypes are formed due to depletion of fusion proteins by translocation from the nucleus, rather than by degradation. Thus, our results show that ribosomal anchoring of Spt8 and TBP is highly effective for depletion in *C. albicans*, indicating that our anchor-away toolbox could be employed for studies with essential as well as non-essential genes in *C. albicans*.

We anticipate that the AAT may not be an optimal inactivation technique in cases where the protein function could be impacted due to the introduction of the FRB-tag to the target proteins. Thus, the full scope of the anchor-away technique would be understood only as more genes are tested by the AAT. Notwithstanding, the AAT holds significant advantages over other gene inactivation methods, especially for *C. albicans*. As demonstrated here for the *C. albicans* TBP (Fig. 4), the AAT permitted rapid depletion without the degradation of TBP. Moreover, anchoring TBP in *S. cerevisiae* led to a substantial transcriptional activation defect at the *GAL1* gene (14), indicating a direct functional impact uncovered by the anchor-away technique. Besides, for mutants that display complex phenotypes such as the constitutive filamentous phenotype of the *spt8*Δ/*spt8*Δ mutant (Fig. 3B), the mutant cells can be processed for downstream molecular characterization without the attendant difficulty of handling filamentous cell populations. Thus, the anchor-away mutants would maintain normal cellular physiology until rapamycin addition, thus limiting any indirect effects caused by permanent gene deletions, including any undesirable aneuploidies widely reported in *C. albicans* (45, 46). Moreover, the AAT can also be effectively used for genes that function under non-permissive conditions, such as at 37°C for temperature-sensitive mutants by rapid depletion. Furthermore, as the candidate genes would be expressed from their native promoter, the effect of depletion can be studied under the physiological level of proteins. Unlike other conditional inactivation mutants such as promoter repression systems or the auxin-regulated degron strains, the anchor-away proteins would be relocated. Our anchor-away system will be a valuable arsenal for high-throughput analysis by systematic construction of anchor-away alleles of each ORF in *C. albicans* followed by phenotypic and molecular characterization to test genome-wide gene functions. Moreover, the AA toolbox developed here can easily be deployed in other CTG clades of pathogenic fungi with auxotrophic markers or by the use of dominant selectable markers (47, 48).

## MATERIALS AND METHODS

### Media and growth conditions

*C. albicans* strains were cultured in yeast extract-peptone (YP)-rich medium for primary/secondary culture, growth assays, and preparation of the protein lysate. All media were supplemented with 20 g/L of either glucose or maltose as required.

### Strains, plasmids, and oligonucleotides

The *C. albicans* parental strains SN152 and SN95 and other strains used in this study are listed in Table S1 (see Table S1 at https://www.jnu.ac.in/Faculty/natarajan/data.htm). The list of plasmids and oligonucleotides in this study is provided in Tables S2 and S3 (see Tables S1 and S2 at https://www.jnu.ac.in/Faculty/natarajan/data.htm).

### Construction of *TOR1-1* and *TOR1-2* strains

To construct the *TOR1-1/TOR1* and *TOR1-2/TOR1* mutant strains, the long oligos ONC981 and ONC982 were designed to introduce A-to-C change (*TOR1-1*) and G-to-T change (*TOR1-2*), respectively, following the strategy reported previously (36). The integration of these oligonucleotides at the genomic *TOR1* locus would also destroy the NheI site in the *FRB* domain of the *TOR1* coding sequence (Fig. 1A). The rapamycin-resistant strains named PSC1 (*TOR1-1/TOR1*) and PSC2 (*TOR1-2/TOR1*) were obtained, and PSC2 was selected for further genetic manipulations.

### Construction of CUG codon-adapted 2×FKBP12

The CUG codon-adapted *2×FKBP12* coding sequence was constructed in multiple steps as follows: first, the gel-purified long oligonucleotide pairs ONC1033/ONC1034, ONC1035/ONC1036, and ONC1037/ONC1038 were used as primers to amplify the *2×FKBP12* coding sequence using plasmid p30582 as the template and Phusion DNA polymerase, and three fragments were prepared. Next, overlap extension PCR (37) was used to fuse the three fragments, and the resulting fragment was used as a template and PCR-amplified using the primer pair ONC1033/1038, and the blunt fragment was cloned into plasmid Ip27 (49) that was digested with Acc65I and end-filled with Klenow DNA polymerase, and plasmid pYPC36 was obtained. Next, the *ACT1* termination sequence was amplified from Ip27 using primers ONC1135 and ONC1136 and cloned into pYPC36. The clones were screened by double digestion with StuI and XhoI and further confirmed by DNA sequencing using ONC1147, and the plasmid pBB24 was obtained. The plasmid pBB24 was further modified to add a 21-bp spacer sequence from p30582 at the 5′ end of *2×FKBP12* by PCR using ONC12011 and ONC12012 and pBB24 as the template, and plasmid pBB43 was obtained. The integration of the spacer sequence at the 5′ end of *2×FKBP12* was confirmed by DNA sequencing.

### Construction of the CUG codon-adapted FRB/mTOR domain

Two sets of purified long oligos ONC1028/ONC1029 and ONC1030/ONC1031 containing the CTG codon changed to CTC (except in ONC1031) were used to produce FRB sequence as fragments 1 and 2 by Klenow DNA polymerase-mediated mutually primed extension of the long oligonucleotides. A diagnostic ApaI site was also introduced in the overlapping 13-bp region in primers ONC1029 and ONC1030 to aid in downstream analysis of recombinant clones. Next, fragments 1 and 2 were again used for overlap extension using Klenow DNA polymerase and further amplified by PCR using primers ONC1028/ONC1031 to generate the blunt-ended *FRB* coding sequence with optimized CTG codons.

The plasmid vectors pSN52 and pSN69 (38) were digested with BamHI and blunt-ended with Klenow DNA polymerase, and the codon-adapted FRB sequence was cloned to produce plasmids pBB2 and pBB8. The correct clones were identified by the presence of the diagnostic ApaI site and further sequenced to confirm the CTG codon changes and that no additional base changes were introduced during the manipulation steps. To introduce a stop codon at the 3′ end of the *FRB* sequence, the primer set ONC1137 and ONC1138 was used to PCR-amplify the *FRB* sequence from pBB8, digested with HindIII and SacI enzymes, and cloned into the corresponding sites in pBB2 to obtain plasmid pBB9 and confirmed by DNA sequencing. Next, to add the *ACT1* terminator sequence in pBB9, the sequence was amplified from Ip27 using the primer pair ONC1139 and ONC1140 and inserted between the SacI and BamHI sites in pBB9. The resulting plasmid pBB28 was confirmed by restriction digestion and DNA sequencing. The *FRB-ACT1t* fragment was PCR-amplified from pBB28 using the primers ONC1141 and ONC1142, digested with BamHI and SpeI enzymes, and cloned into pBB8 digested with the same enzymes, and the resulting plasmid pBB30 was confirmed by DNA sequencing.

### Construction of anchor-away base strain

To construct *C. albicans* strain BB13, we first deleted one allele of *RBP1* in *C. albicans* strain SN152 using the deletion cassette amplified from Ip27 as a template using Phusion DNA polymerase and primer pairs ONC1026-ONC140 and ONC1027-ONC141 bearing 1.4-kb overlapping region and homology to the upstream and downstream regions of *RBP1* ORF. The resulting *SAT1* gene split fragment mixture was transformed into the strain SN152, and Nou^R^ colonies were selected and screened for the correct integration by PCR using the gene-specific upstream primer ONC1000 and the cassette-specific primer ONC140 and candidates were identified, and the resulting strain *rbp1*∆*::SAT1-FLP/RBP1* was named BB13.

Next, we deleted one allele of *RBP1* in the *TOR1-2/TOR1* strain PSC2 using the same strategy as described above for strain BB13 with the following exception. To increase homology to the *RBP1* locus, the *SAT1-FLP* cassette was amplified from the genomic DNA of BB13 (*rbp1*∆*::SAT1-FLP*) using ONC1122 and ONC140 and ONC1123 and ONC141 that contained 486 bp and 719 bp homology, respectively, to the upstream and downstream regions of *RBP1* ORF. The transformants were selected on YPD plates containing Nou, and the correct integration was screened by PCR using primer pair ONC1125 and ONC141, and the strain BB10 (*rbp1*∆*:: SAT1-FLP/RBP1 TOR1-2/TOR1*) was obtained. Next, to obtain the Nou^S^ derivative of BB10, the strain was grown for 8 hours in YPD medium containing maltose to allow *FLP*-mediated excision of the *SAT1* flipper cassette. The resulting Nou^S^ strains were verified for the excision of the cassette by PCR using primers ONC1000 and ONC 140, and the correct strain was named BB21 (*rbp*Δ*:: FRT/RBP1 TOR1-2/TOR1*). The second *RBP1* allele in BB21 was deleted as mentioned previously, and the absence of the *RBP1* ORF in the resulting strain was confirmed by PCR using ORF-specific primers ONC1001 and ONC1002, and the homozygous *rbp1*∆/*rbp1*∆ strain BB29 strain was obtained. Finally, the Nou^S^ derivative of strain BB29 was selected and named BB37 (*rbp1*∆*::FRT/rbp1*∆*::FRT TOR1-2/TOR1*) and was used for further manipulations.

Next, the *2×FKBP12* sequence tag was added to the *RPL13* 3′-terminal region by the inclusion of a spacer sequence from plasmid pBB43 as follows. The *2×FKBP12-SAT1* cassette was amplified from plasmid pBB43 in two fragments, the up-split (ONC12018-ONC140) and down-split (ONC141-ONC1133), and transformed into *C. albicans* BB37 and Nou^R^ transformants obtained, and the in-frame integration of *2×FKBP12* tag-bearing spacer at the 3′ end of *RPL13* was confirmed using ONC1134-ONC1136, and the anchor-away base strain BB85 was obtained.

### Construction of *CaSPT8-FRB* and *CaTBP1-FRB* anchor-away *C. albicans* strains

The anchor-away strains were constructed in two stages. First, one allele of *SPT8* and *TBP1* was deleted to make heterozygous strains, followed by epitope-tagging of each of the second allele with the *FRB* sequence. To delete the first allele, the deletion cassette was PCR-amplified using primer sets ONC12022/ONC12021 for *SPT8* or ONC12023/ONC1149 for *TBP1* using pSN40 as a template, and the DNA was transformed into strain BB85, and leucine prototrophs were selected. The correct clones were identified by PCR using primers ONC33 and either ONC12025 (for *SPT8*) or ONC 1151 (for *TBP1*), and resulting strains BB114 and BB107 were obtained. The *SAT1* marker was flipped out and confirmed by PCR using primers ONC140/141, and heterozygous deletion strains BB120 and BB119 were obtained.

Next, the second allele of *SPT8* and *TBP1* was tagged with the *FRB* sequence at the carboxyl terminus using PCR amplicons obtained using primers ONC12020/ONC12021 (*SPT8*) and ONC1148/ONC1149 (*TBP1*) and pBB30 as a template and transformed into BB120 and BB119. The Arg^+^ Leu^+^ transformants were selected and directly screened for depletion by assessing the altered morphology (*SPT8*) or lethality (TBP) in the presence of rapamycin (1 µg/mL). The mutants that showed the phenotype were verified for the correct integration of the *FRB-ARG4* cassette by PCR using ONC12024/ONC12025 for *SPT8* or ONC1150/ONC1151 for *TBP1,* and the anchor-away strains BB122 (*SPT8-FRB*) and BB123 (*TBP-FRB*) were obtained.

### Preparation of whole-cell extracts and Western blotting

*C. albicans* and *S. cerevisiae* strains were pre-grown in YPD overnight and diluted to 25 mL fresh media to ~0.1 OD_600_. About 50 OD_600_ cells were harvested, washed with cold sterile water, and resuspended in 150 µL ice-cold Winston buffer (50) (10% glycerol, 40 mM HEPES-NaOH [pH 7.5], 350 mM NaCl, and 0.1% Tween 20) containing protease inhibitors (2.5 µg/mL aprotinin, 2 mM benzamidine, 1 mM dithiothreitol, 2 µg/mL leupeptin, 2 µg/ml pepstatin, 100 mM phenylmethylsulfonyl fluoride, 10 µg/mL tosylsulfonyl phenylalanyl chloromethyl ketone, and 10 µg/mL *N-p*-tosyl-L-lysine chloromethyl ketone). Cell lysis was carried out with glass bead vortexing at 4°C, and the cleared whole cell extract was obtained by centrifugation at 13,000 rpm for 15 minutes at 4°C as described before (49). Total protein concentrations in cell lysates were estimated by using the Bio-Rad protein assay reagent, and ~200 µg total protein was separated on SDS-polyacrylamide gels and transferred to nitrocellulose membranes (Protran Premium 0.45 µm nitrocellulose; GE Healthcare). Anti-FKBP12 (Novas Biologicals), anti-FRB (Enzo Bioscience), and anti-G6PD (Sigma/Merck) antibodies were used to probe membranes with the horseradish peroxidase (HRP)-conjugated anti-rabbit secondary antibody and detected with ECL Prime Western Blotting Detection Reagent (GE Healthcare). The blot was exposed at different times as required to a Hyperfilm ECL X-ray film (GE Healthcare).

### Viability after rapamycin treatment

The various strains were pre-grown for 14–16 hours in YPD (2 mL), and each culture was started with optical densities (OD_600_) of 0.1 by dilution in a fresh YPD (10 mL) medium. All cultures were grown for approximately 6 hours at 30°C till their OD_600_ reached between 0.8 and 1.5, and ~2.0 OD_600_ cells were harvested and resuspended in 2 mL fresh YPD. To one set of cultures, rapamycin was added to a final 1 µg/ml, and equivalent volume DMSO (vehicle control) was added to the duplicate culture and allowed to grow at 30°C, and about 200 µl cultures (treated and untreated) were harvested at 0, 15, 30, 90, and150 minutes of rapamycin addition. The cells were serially diluted 10-fold and spotted onto YPD plates, which were incubated at 30°C and viability recorded for several days.

## Data Availability

All materials generated in this work would be made freely available to the academic community.
